# Risk of invasive waterfowl interaction with poultry production: Understanding potential for avian pathogen transmission via species distribution models

**DOI:** 10.1002/ece3.11647

**Published:** 2024-07-18

**Authors:** Reilly T. Jackson, Percival M. Marshall, Chris Burkhart, Julia Schneck, Grant Kelly, Caleb P. Roberts

**Affiliations:** ^1^ Department of Biological Sciences University of Arkansas Fayetteville Arkansas USA; ^2^ U.S. Geological Survey, Arkansas Fish and Wildlife Cooperative Research Unit University of Arkansas Fayetteville Arkansas USA

**Keywords:** Egyptian Goose, human‐wildlife interaction, Mute Swan, poultry, species distribution modeling

## Abstract

Recent outbreaks of highly pathogenic avian influenza have devastated poultry production across the United States, with more than 77 million birds culled in 2022–2024 alone. Wild waterfowl, including various invasive species, host numerous pathogens, including highly pathogenic avian influenza virus (HPAIV), and have been implicated as catalysts of disease outbreaks among native fauna and domestic birds. In major poultry‐producing states like Arkansas, USA, where the poultry sector is responsible for significant economic activity (>$4 billion USD in 2022), understanding the risk of invasive waterfowl interactions with domestic poultry is critical. Here, we assessed the risk of invasive waterfowl‐poultry interaction in Arkansas by comparing the density of poultry production sites (chicken houses) to areas of high habitat suitability for two invasive waterfowl species, (Egyptian Goose [*Alopochen aegyptiaca*] and Mute Swan [*Cygnus olor*]), known to host significant pathogens, including avian influenza viruses. The percentage of urban land cover was the most important habitat characteristic for both invasive waterfowl species. At the 95% confidence interval, chicken house densities in areas highly suitable for both species (Egyptian Goose = 0.91 ± 0.11 chicken houses/km^2^; Mute Swan = 0.61 ± 0.03 chicken houses/km^2^) were three to five times higher than chicken house densities across the state (0.17 ± 0.01 chicken houses/km^2^). We show that northwestern and western Arkansas, both areas of high importance for poultry production, are also at high risk of invasive waterfowl presence. Our results suggest that targeted monitoring efforts for waterfowl‐poultry contact in these areas could help mitigate the risk of avian pathogen exposure in Arkansas and similar regions with high poultry production.

## Graphical abstract

Across the United States, poultry production can be a major economic activity; however, this industry is threatened by the introduction of waterfowl‐borne pathogens such as highly pathogenic avian influenza. Here, we assessed the risk of invasive waterfowl‐poultry interactions in Arkansas by comparing the density of poultry production sites (chicken houses) to areas of high habitat suitability for two invasive waterfowl species (Egyptian Goose [*Alopochen aegyptiaca*] and Mute Swan [*Cygnus olor*]) known to host significant pathogens. We found that these invasive waterfowl are spatially aggregated in regions where poultry density is 3–5 times higher than the state average and their distribution is largely driven by the prevalence of urban land cover.

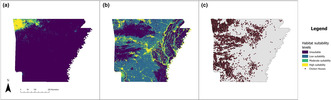

## 1 INTRODUCTION

Several waterfowl (Order: Anseriformes) species are highly successful invaders that can host and transmit pathogens, such as highly pathogenic avian influenza virus (HPAIV), avian orthoavulavirus‐1 (formerly Newcastle Disease virus; NDV), and numerous harmful bacteria and parasites (Clark, 2003; Fallacara et al., 2004; Pedersen et al., 2014). These pathogens can cause extensive mortality in non‐reservoir native and domestic avian species, thereby threatening both biodiversity and food security (Alders et al., 2014; Ayala et al., 2020; Ramey et al., 2022). Surveillance efforts have identified the presence of HPAIV, NDV, and many enteric bacteria in invasive waterfowl in North America (Brown & Bevins, 2017; Kistler et al., 2012; Smith et al., 2020; Turpin et al., 2008). For example, ducks (Family: Anatidae) are the natural reservoir of several avian influenza viruses and have been heavily implicated in the global spread of HPAIV due to their long migrations (Blagodatski et al., 2021; Olsen et al., 2006). During stopovers, migratory ducks will use agricultural lands simultaneously occupied by domestic poultry, creating an interface for poultry exposure to waterfowl‐borne pathogens (Keawcharoen et al., 2011; Naguib et al., 2019). Similarly, invasive waterfowl, such as the Egyptian Goose (*Alopochen aegyptiaca*; family: Anatidae), have also been documented aggregating and residing in agricultural lands for extended periods and may subsequently expose resident poultry to their pathogens when sharing these spaces (Elmberg et al., 2017; Fox et al., 2017; McDuie et al., 2022; Smith & James, 2012).

The potential proximity between invasive waterfowl and poultry production—and subsequent sharing of resources such as feed and water bodies—may facilitate domestic poultry exposure to pathogens shed by co‐occurring waterfowl species (Ayala et al., 2020; Navarro‐Gonzalez et al., 2020; Plowright et al., 2017). The development and growth of commercial poultry production entails dense populations of birds, frequent vaccination, and antibiotic application to maintain animal health and a constant turnover of individuals that results in naïve birds circulating continuously through these systems (Mottet & Tempio, 2017). Together, these factors sustain bird populations highly susceptible to pathogen infection, with density and immunological naivety further driving large‐scale outbreaks (Biggs, 1982; Leibler et al., 2009; Sharma, 1999). To prevent outbreaks, biosecurity measures aimed at preventing or reducing the risk of pathogen exposure in these flocks are implemented to keep production viable (United States Department of Agriculture [USDA], 2005). For example, many biosecurity measures focus on exclusion and are designed to reduce the risk of infectious fomite introduction into secure areas housing poultry, such as through wild bird feces or secretions on equipment (USDA, 2013). However, despite these efforts, biosecurity measures often fail, as evidenced by waterfowl‐borne disease outbreaks in commercial poultry worldwide (Elmberg et al., 2017; Guinat et al., 2020; Newell et al., 2011; Rehan et al., 2019). As the poultry sector continues to grow, failures in these biosecurity measures may increase in kind, presenting repeated economic and biological losses to producers (Ayala et al., 2020).

For these reasons, there is a critical need to identify potential areas of contact between invasive waterfowl species that carry lethal pathogens and domestic poultry producers. Outbreaks of avian pathogens in poultry have negatively impacted economic activity, as seen in the 2022–2024 outbreak of HPAIV in North America (Ayala et al., 2020; USDA, 2024). Throughout the southeastern United States, poultry production is a major agricultural product and contributor to state economies. In Arkansas, poultry production was responsible for more than $4 billion USD in revenue in 2022, making this sector one of the most important industries in the state (Arkansas Department of Agriculture, 2022). However, the recent avian influenza outbreak has led to massive flock culls, with over 77 million birds culled in the US during 2022–2024 in response to the outbreak, resulting in severe economic effects (USDA, 2024).

Here, our overall goal was to assess the risk for invasive waterfowl‐poultry contact by comparing the overlap between invasive species' habitat suitability and areas of dense poultry production in Arkansas. We defined poultry production density as the density of commercial chicken houses. To accomplish this, we developed species distribution models (SDMs) for two invasive waterfowl species, the Egyptian Goose and the Mute Swan (*Cygnus olor*), both of which are recent invaders of Arkansan agricultural lands (late 1980s; eBird, 2022; Smith & James, 2012). These species may host and transmit several avian pathogens, including avian influenza viruses and NDV (Burger et al., 2012; Pedersen et al., 2014; Pfitzer et al., 2000). Our specific objectives were to use the generated SDMs to (1) identify bioclimatic and land cover drivers of environmental suitability for each invasive waterfowl species in Arkansas and (2) quantify the mean density of poultry production in Arkansas and compare that to poultry production density in areas highly suitable for invasive species.

## 2 METHODS

### 2.1 Study area

Our study area was the state of Arkansas, located just west of the Mississippi River in the United States. Arkansas covers ~137,860 km^2^ and dominant land cover types include agriculture, lowland hardwood forest, savannah, and upland hardwood and pine forests. Arkansas has a humid‐subtropical climate, characterized by heavy rainfall and large variations in temperatures throughout the year (Runkle et al., 2022). Summers are hot and humid, and winters are short and cool. Elevation across the state ranges from 17 to 839 m, and temperature typically varies according to this elevational gradient.

### 2.2 Data collection

Our study focused on the distribution and habitat suitability of two invasive waterfowl species, the Egyptian Goose and the Mute Swan. We chose these species for four reasons. First, these species are recent invaders of Arkansas (eBird, 2022; Smith & James, 2012). Second, these species have noted negative impacts on native species in areas of their invaded ranges (Gayet et al., 2020; Huysentruyt et al., 2020). Third, these species can host and transmit avian pathogens of concern to poultry (Burger et al., 2012; Pedersen et al., 2014; Pfitzer et al., 2000). Lastly, we wanted to further understand potential impacts of these invasive species in Arkansas. We collected presence data for our two focal invasive waterfowl species for 2014–2022 from the eBird data portal. We limited our presence data to points collected within 5 years of the 2019 Copernicus Global Landcover publication year (explained below). Evidence shows that at least one of our focal species, the Egyptian Goose, has shifted its tolerance for thermal extremes in invaded areas, which facilitates the selection of habitat significantly different than habitat in its home range (Marshall, 2023). Due to this niche shift, we focused our SDMs on the invaded range in Arkansas and included records with a 200 km buffer around the state to accurately identify distributions in the invaded area (Jarnevich et al., 2022; Nikkel et al., 2023; Figure S1).

To model the distribution of poultry production in the state, we used chicken house data reported by the Arkansas Highway and Transportation Department (AHTD, 2014). In brief, this point data represent commercial poultry house facilities identified and mapped via satellite imagery. Animals housed in these buildings may include chicken, turkey, or duck, and may represent broiler or egg‐laying production. While this dataset may not incorporate the diversity of poultry production in Arkansas, such as backyard flocks, this is the most comprehensive, publicly accessible data for the state.

### 2.3 Analysis

#### 2.3.1 Environmental suitability for Egyptian Goose and Mute Swan

We used the Maximum Entropy program (MaxEnt, Version 3.4.4) to model the environmental suitability of our focal species within the state of Arkansas. MaxEnt is a popular method for ecological niche modeling because of its model stability and predictive performance using presence‐only data, even with small sample sizes (Phillips & Dudík, 2008; Saupe et al., 2015). This program uses environmental variables and species occurrence information to calculate geographic constraints and develop distribution patterns within modeled constraints, ultimately predicting habitat suitability for a particular species within a study area (Merow et al., 2013; Phillips et al., 2006).

To model species distributions, we used several environmental variables due to their inherent restrictions on species presence (Anderson et al., 2011). We evaluated 19 bioclimatic variables (BIO1–BIO19) and mean elevation from the WorldClim V2 Database with a spatial resolution of 30 s (~1 km^2^; www.worldclim.org; Fick & Hijmans, 2017). We also incorporated the cover fraction raster layers of the 2019 Copernicus Global Landcover dataset (~100 m^2^ resolution; Buchhorn et al., 2020) in models. We resampled all percent cover landcover raster layers to 1 km^2^ resolution using the nearest neighbor function in ArcGis Pro 2.9 (ESRI, Redlands, California, USA) to match the resolution of our bioclimatic variables. Due to the high correlation among variables, we used a raster correlation analysis for all 30 elevation, bioclimatic, and landcover raster layers clipped to our 200 km buffer around Arkansas to test for correlation (“raster” package, Hijmans et al., 2022). We used a correlation coefficient of 0.7 as a cut‐off for determining correlation, which left us with elevation, 8 landcover variables, and 8 bioclimatic variables to use within our models (Table 1; Lake et al., 2020).

**TABLE 1:** Climatic, topographic, and landcover variables included in Maximum Entropy (MaxEnt) species distribution modeling of two invasive waterfowl species in Arkansas.

Included variable	Definition
BIO2	Mean Diurnal Temperature Range
BIO4	Temperature Seasonality
BIO5	Maximum Temperature of Warmest Month
BIO8	Mean Temperature of Wettest Quarter
BIO11	Mean Temperature of Coldest Quarter
BIO15	Precipitation Seasonality
BIO16	Precipitation of Wettest Quarter
BIO18	Precipitation of Warmest Quarter
Elevation	Mean elevation
Urban	Percent ground cover for urban land cover class
Bare Cover	Percent vegetation cover for bare‐sparse‐vegetation land cover class
Crops	Percent vegetation cover for cropland land cover class
Grass	Percent vegetation cover for herbaceous vegetation land cover class
Moss	Percent vegetation cover for moss and lichen land cover class
Seasonal Water	Percent ground cover for seasonal water land cover class
Shrubland	Percent vegetation cover for shrubland land cover class
Permanent Water	Percent ground cover for permanent water land cover class

*Note*: All variables are sampled at 1 km^2^. BIO2–BIO15 and elevation were sourced from the WorldClim Database and landcover data was sourced from the 2019 Copernicus Global Landcover Layers.

Given that our species occurrence data were sourced from citizen science initiatives, uneven sampling often leads to geographic bias of species presence (Robinson et al., 2017). Sampling bias can lead to spatial extrapolation errors in species distribution models, leading to inaccurate model outputs (Raes & ter Steege, 2007). To correct for sampling bias, we created a bias file for our focal species (“raster” package; Hijmans et al., 2022). A bias raster file indicates relative sampling effort and provides a priori relative sampling probabilities. Bias files can reduce estimation error caused by the over‐fitting of environmental variables in highly surveyed areas and improve the overall model performance (Kramer‐Schadt et al., 2013; Phillips et al., 2009). To create our bias file, we rasterized our occurrence data within the study area and used kernel density estimates to identify regions with high concentrations of occurrence points (“MASS” package; Ripley et al., 2023). Pseudo‐absences required by MaxEnt to model species distribution were then preferentially selected from these regions to account for the spatial bias of our data.

To further optimize model generation, we adjusted model settings instead of using default MaxEnt parameters (Merow et al., 2013). We analyzed different arrangements of feature combinations and regularization multipliers of parameters to allow MaxEnt to best infer species' response to environmental factors mathematically. We generated background data points based preferentially around areas of high sampling effort indicated in our bias files for each species. We used the “randomkfold” method of cross‐validation for analyses with 10 repetitions. We analyzed five feature combination parameters (linear [L], quadratic [Q], hinge [H], product [P], and threshold [T]), and all combinations of these parameter types in conjunction with a regularization multiplier to identify the best model parameter selection based on lowest Aikake's information criterion (AIC) values (range: 1–5; “ENMeval” package, Kass et al., 2021). All model correlations and optimizations were performed in R version 4.1.3.

Using outputs from preliminary model optimization analyses, we changed input feature combinations and regularization multipliers for each species. In Maxent, we used the “cross‐validate” replicated run type where the occurrence data were randomly split into 10 equal‐sized “folds” and excluded “folds” were used for model evaluation. We included our bias raster file for each species within analyses and our environmental variables. Model accuracy was determined by area under the operating curve (AUC) values. We created habitat suitability maps for analysis based on the mean prediction of our cross‐validated MaxEnt models for each species. We assigned habitat suitability classes to cells based on the predicted probability of occurrence. Raster cells with values 0–0.2, or a <20% probability of occurrence, were unsuitable areas. Cells valued at 0.2–0.4, or a 20%–40% probability of occurrence, were areas with low suitability. Areas with 0.4–0.6 values, i.e., a 40%–60% probability of occurrence, were moderate, and values >0.6, with a >60% probability of occurrence, were defined as areas with high suitability for species distribution (Convertino et al., 2014).

#### 2.3.2 Quantification and comparison of poultry production density in areas highly suitable for invasive waterfowl presence

We used ArcGIS Pro 2.9 to calculate the spatial extent of each habitat suitability category for each species within Arkansas. We identified the number of chicken houses within each habitat suitability category and the density of chicken houses per square kilometer to understand where areas of high habitat suitability overlapped with areas of high chicken house density. Density of chicken houses was calculated by taking the number of chicken houses within a habitat suitability cell and dividing by the total area of the cell (km^2^).

To see if chicken house density in areas of high habitat suitability for our focal species was greater than randomly expected, we measured the density of chicken houses across Arkansas to compare to chicken house density within areas of high suitability for each species. Based on the resolution of our spatial data (~1 km^2^), we used a 1 km^2^ grid over the state of Arkansas to identify the variation in density of chicken houses across the state. Our data were not normally distributed (Anderson‐Darling test; *p* < .001). Therefore, we used a Kruskal–Wallis test with a Wilcoxon test for pairwise comparisons to identify differences in chicken house density in highly suitable areas for our two focal species and across Arkansas. We then calculated the 95% confidence intervals of the density of chicken houses within areas of high suitability for our focal species and compared these to the general density of chicken houses across Arkansas.

## 3 RESULTS

We obtained 1739 occurrence points for our two focal invasive waterfowl species and 21,477 chicken houses (Table 2). The mean test AUC after 10 repetitions was 0.93 and 0.86 for our Egyptian Goose and Mute Swan models, respectively.

**TABLE 2:** The number of input points (occurrences), the total area of highly suitable habitat in Arkansas (identified via Maximum Entropy program, MaxEnt), the total number of chicken houses located within highly suitable areas of Arkansas, and the mean density (the number of chicken houses per km^2^; ±95% confidence interval) of chicken houses within highly suitable areas of two invasive waterfowl species in Arkansas.

Species	No. occurrences	Total area (km^2^)	No. chicken houses	Mean chicken house density
Egyptian Goose	463	4142.77	4927	0.91 ± 0.11
Mute Swan	1276	12,144.20	2026	0.61 ± 0.03

### 3.1 Environmental suitability for Egyptian Goose and Mute Swan

**FIGURE 1:**
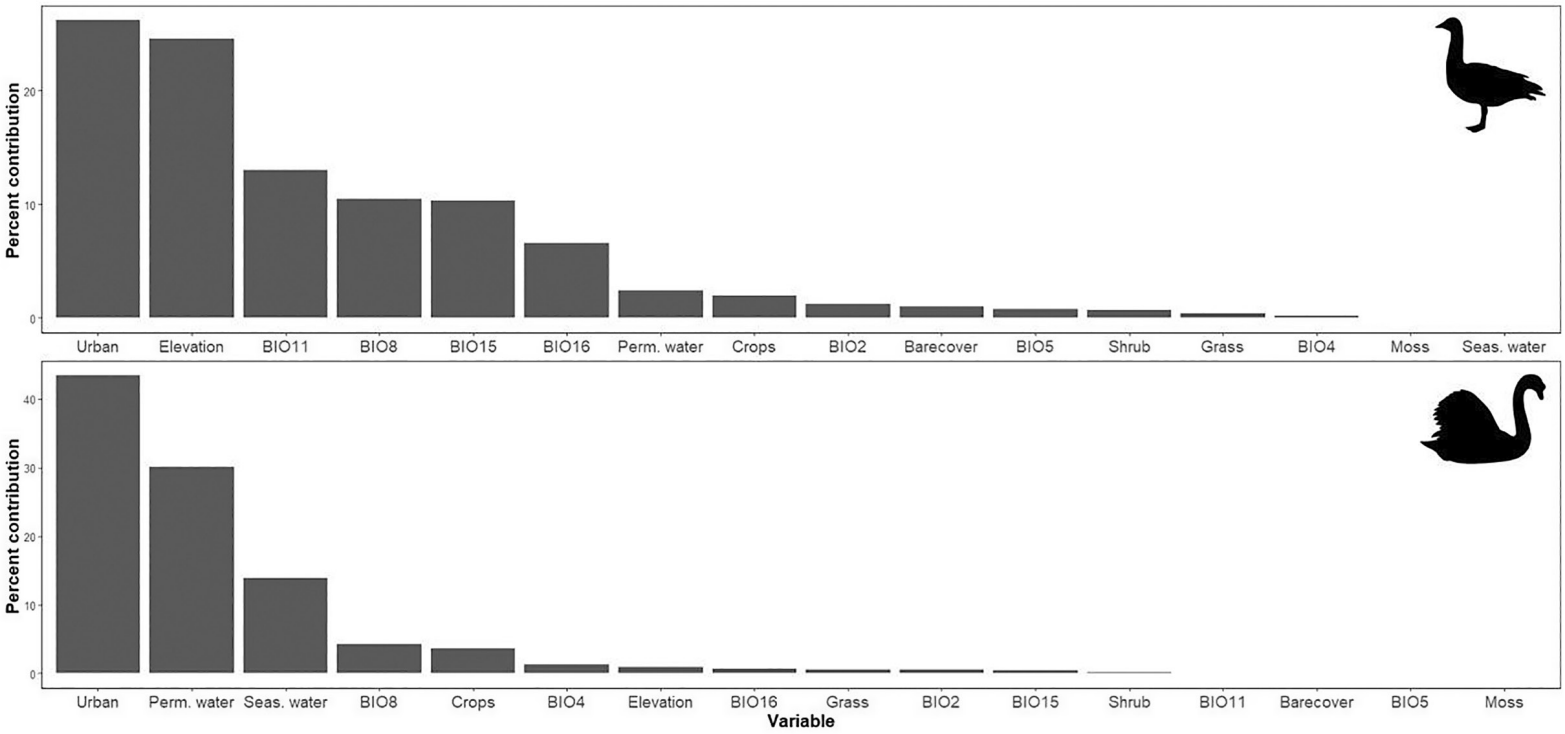
Variable contribution to species distribution models for Egyptian Goose (*Alopochen aegyptiaca*; upper panel) and Mute Swan (*Cygnus olor*; lower panel) in Arkansas, USA. BIO2–BIO15 and elevation were sourced from the WorldClim Database and landcover variables were sourced from the 2019 Copernicus Global Landcover Layers. Animal silhouettes are from PhyloPic.

**FIGURE 2:**
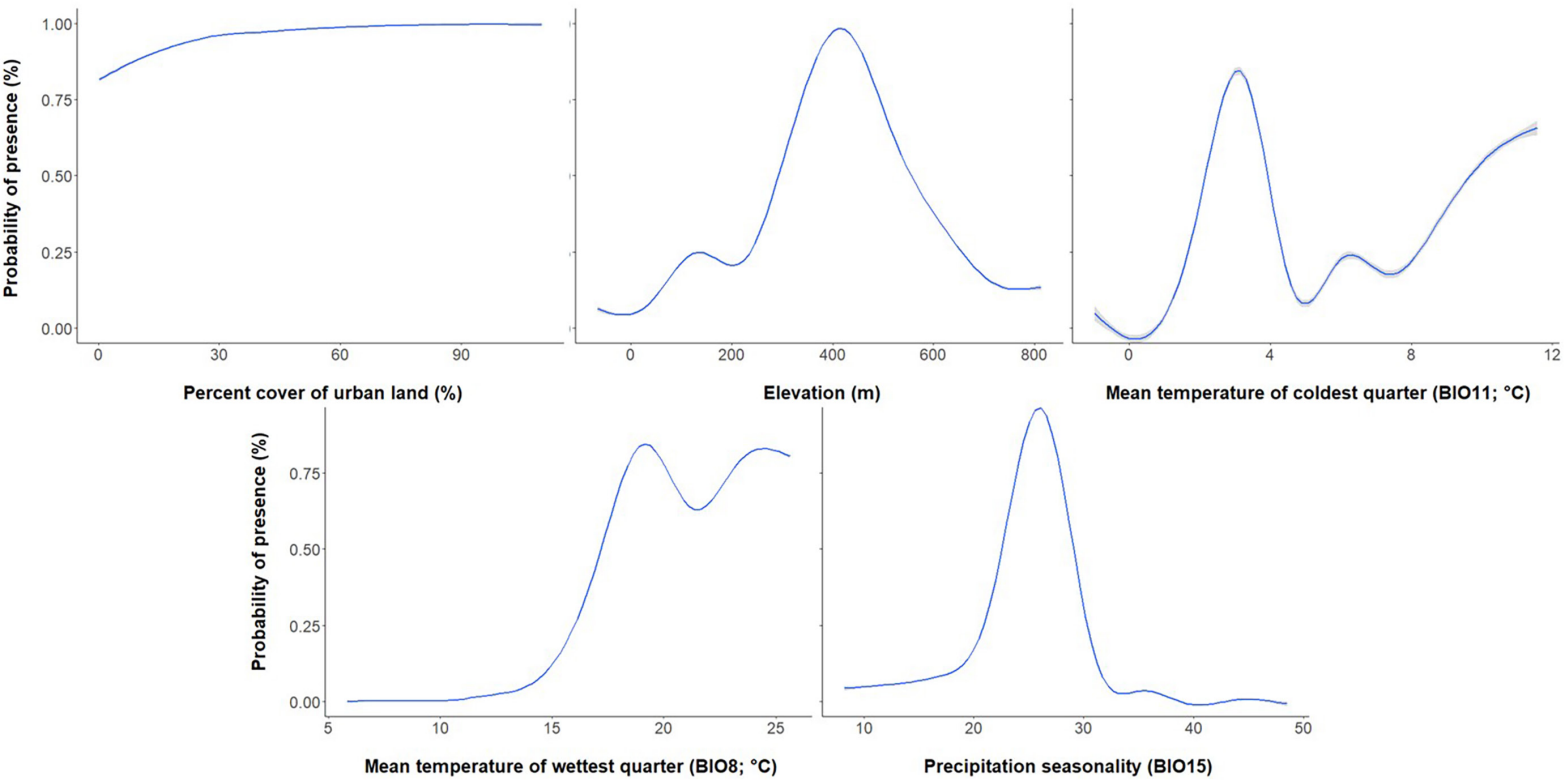
Egyptian Goose (*Alopochen aegyptiaca*) habitat suitability response curves to the highest contributing landcover and bioclimatic variables for Arkansas, USA. BIO8–BIO15 and elevation were sourced from the WorldClim Database and landcover variables were sourced from the 2019 Copernicus Global Landcover Layers.

**FIGURE 3:**
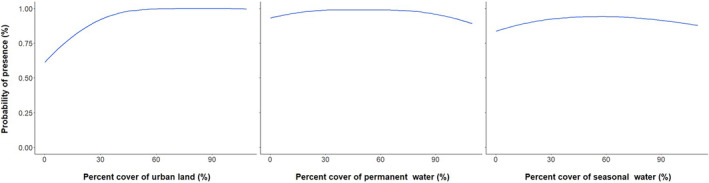
Mute Swan (*Cygnus olor*) habitat suitability response curves to the highest contributing landcover variables for Arkansas, USA. Landcover variables were sourced from the 2019 Copernicus Global Landcover Layers.

**FIGURE 4:**
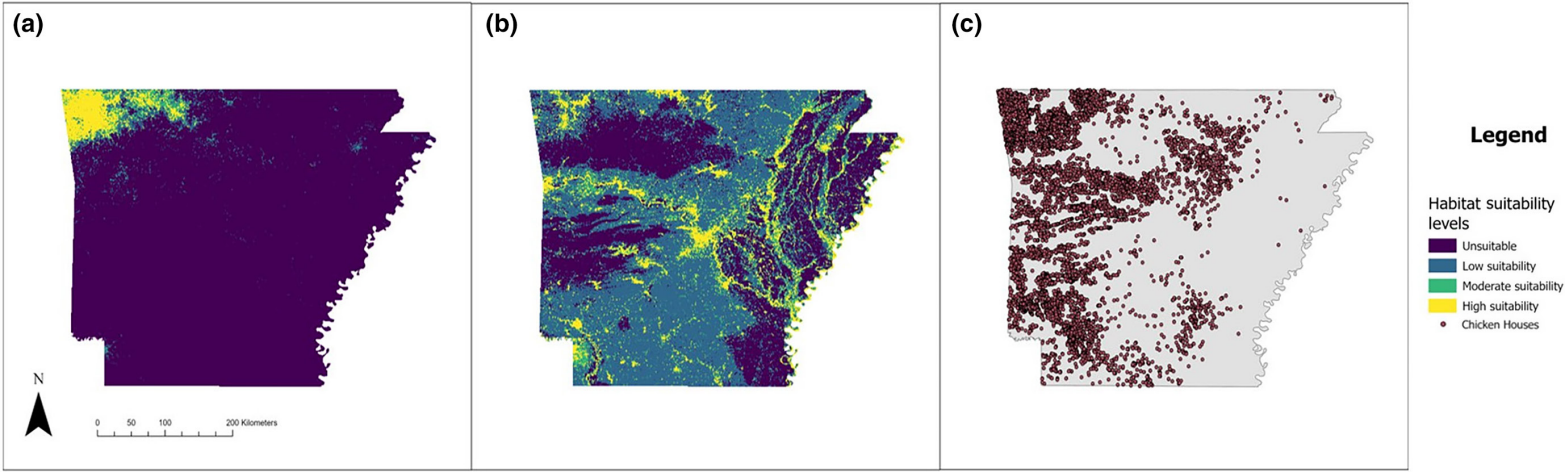
Predicted habitat suitability maps for (a) Egyptian Goose (*Alopochen aegyptiaca*) and (b) Mute Swan (*Cygnus olor*) in Arkansas, USA. Panel (c) shows the distribution of chicken houses within Arkansas. Chicken house data sourced from the Mapping Section of the Planning and Arkansas State Highway and Transportation Department's Research Division, in cooperation with the U.S. Department of Transportation (AHTD, 2014).

Percent cover of urban areas was the most important contributing factor for both Egyptian Goose (*n* = 26.2% contribution) and Mute Swan (*n* = 43.4% contribution), with development increasing the likelihood of habitat suitability for both species (Figures 1, 2, 3). Environmental variables contributing heavily to Egyptian Goose distribution also included elevation (*n* = 24.6% contribution), mean temperature of the coldest quarter (BIO11; *n* = 13% contribution), mean temperature of the wettest quarter (BIO8; *n* = 10.5% contribution), and precipitation seasonality (BIO15; *n* = 10.3% contribution). In turn, Mute Swan distribution was heavily influenced by percent cover of permanent water (*n* = 30.1% contribution) and seasonal water (*n* = 13.9% contribution). All other variables contributed <10%. Highly suitable habitat for Egyptian Goose was largely clustered in northwest Arkansas (Figure 4a). The distribution of highly suitable Mute Swan habitat was more widespread, although western, central, and eastern Arkansas were favored (Figure 4b).

### 3.2 Quantification and comparison of poultry production density in areas highly suitable for invasive waterfowl presence

**FIGURE 5:**
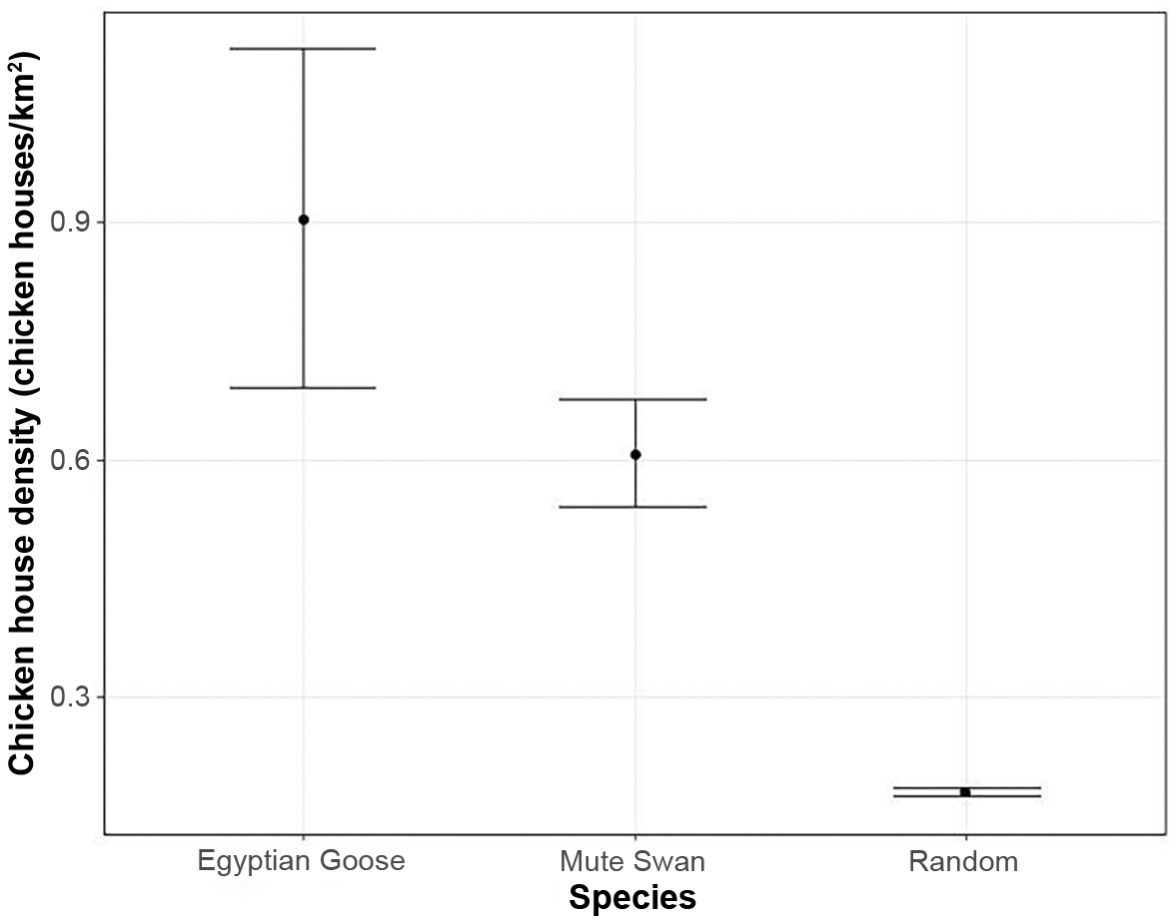
Mean density of chicken houses (chicken houses/km^2^ ± 95% confidence interval) within highly suitable areas for two Arkansan invasive waterfowl species (Egyptian Goose [*Alopochen aegyptiaca*] and Mute Swan [*Cygnus olor*]) and within the state of Arkansas (Random).

Poultry production was most dense in western Arkansas, with chicken houses more dispersed across central Arkansas (Figure 4c). The density of chicken houses within areas of high suitability for both Egyptian Goose and Mute Swan was significantly higher than the mean density of chicken houses across Arkansas (Kruskal–Wallis *χ* ^2^ = 798.73, *p* < .001; Figure 5). Species distribution models showed that highly suitable habitat for Egyptian Goose has 0.91 (±0.11; at 95% confidence) chicken houses/km^2^, which is 5.35 times higher than the mean density of chicken houses across the state of Arkansas (0.17 ± 0.01 chicken houses/km^2^ at 95% confidence; *p* < .001; Table 2; Figure 5). Mean chicken house density in areas of high suitability for Egyptian Goose (0.91 ± 0.11 chicken houses/km^2^ at 95% confidence) was higher than in areas of high suitability for Mute Swans (0.61 ± 0.03 chicken houses/km^2^), but the latter was still 3.58 times higher than the mean density of chicken houses across Arkansas (*p* < .001; Table 2; Figure 5).

## 4 DISCUSSION

We found that areas highly suitable for two invasive waterfowl species known to host avian influenza viruses and NDV had three to five times higher densities of commercial chicken houses than statewide mean chicken house density—in a region where the annual poultry production revenues are billions of USD per year. Egyptian Goose and Mute Swan distributions are most closely associated with urban and water landcover. This association with urban regions, often at a permanent aquatic interface for Mute Swan, could increase the likelihood of contact between invasive waterfowl and domestic fowl as agricultural sites, like poultry production, are characterized by urban land and water cover, typically livestock ponds or similar.

Spatial overlap of livestock and invasive animals is an important driver of interactions between species, which can facilitate spillover of invasive wildlife‐borne pathogens into domestic animals (Plowright et al., 2017; Vilcinskas, 2015). Specifically, our results show that agricultural spaces may be areas where domestic animals, like poultry, may be exposed to invasive waterfowl and their pathogens (Elmberg et al., 2017; Velkers et al., 2021). Many such pathogens, like HPAIV, can be transmitted to poultry through direct bird‐to‐bird contact, poultry contact with a contaminated environment, or through aerosolized secretions from infected waterfowl (Pepin et al., 2023; Rohani et al., 2009; Zhao et al., 2019). Therefore, in areas highly suitable for invasive waterfowl, especially northwest and central Arkansas, curtailing invasive waterfowl presence will likely reduce exposure risk for poultry. Additional pathogen surveillance effort for these species present at the wildlife‐urban interface would also benefit poultry producers operating in these areas.

Landcover was the main predictor of habitat suitability for our two focal species, the Egyptian Goose and Mute Swan. Akin to previous research, our study found that urban landcover contributed the most to defining habitat suitability for these species, with suitability increasing in line with development percent cover (Groom et al., 2020; Petrie & Francis, 2003). Urban landscapes are often highly disturbed regions, with increased human activity facilitating the removal of native species that vacate their ecological niches (Burton et al., 2005). Invasive species can utilize these disruptions by dominating empty niches, allowing their populations to expand unchecked in these novel environments (Gayet et al., 2014; Kornherr & Pütz, 2022). In areas like Arkansas, where urban and agricultural development is increasing, invasive species may follow, thereby creating additional interfaces for waterfowl contact with humans and domestic animals (Boustan et al., 2013; Crowl et al., 2008).

Regional suitability for invasive waterfowl and poultry production in Arkansas are likely interlinked and relate to numerous factors. The cultural and historical reliance of this area on chicken production, the mild climate appropriate for poultry (and waterfowl), and the urbanizing landscape that reduces poultry production and transportation costs, all influence waterfowl distribution and commercial poultry development (Groom et al., 2020; Lord, 1971; Petrie & Francis, 2003; Sambidi, 2003). However, these circumstances are not unique to Arkansas. Poultry production and its supporting factors are equally high in neighboring southeastern states, including Alabama, Georgia, Mississippi, and Texas (USDA, 2023). These states also have populations of invasive waterfowl species, including Egyptian Goose and Mute Swan, invading urban areas (Callaghan & Brooks, 2017; eBird, 2022; Marshall, 2023). Therefore, these states may face similar situations facilitating poultry contact with invasive waterfowl. Work is needed to characterize the potential for poultry‐waterfowl interactions in these states, although the lack of open‐access poultry production data may stall such efforts.

While our study provides important information identifying regions of spatial overlap of poultry production and invasive waterfowl habitat suitability, as well as factors driving that habitat suitability, our analyses have important limits. First, our study utilized waterfowl location data from eBird, which may suffer from sociodemographic bias that is not easily corrected for with current data outputs (Rutter et al., 2021). Second, the chicken house data used in this study, derived from satellite imagery of commercial poultry properties, provided little detail on the poultry type and biosecurity measures employed at these sites, both of which may drive the risk of exposure to waterfowl‐borne pathogens. We additionally were not able to include data on backyard poultry establishments, which may be at increased risk for exposure to wild waterfowl (Gentile et al., 2024). We further limited the focus of our study to recent waterfowl invaders of Arkansas which, while these species may constitute threats, are not the only hosts of concerning poultry pathogens. Therefore, future efforts encompassing a wider scope of waterfowl and utilizing more comprehensive poultry data, potentially involving genetic methods, may further clarify the risk of poultry exposure to waterfowl‐borne pathogens. However, open‐access sources of agricultural data, such as poultry production and associated biosecurity data, are needed if these efforts are to be successful and benefit poultry producers.

Species distribution models are broadly used across ecological disciplines and are critical tools in forecasting pathogen exposure risk for people and domestic animals (Belkhiria et al., 2018; Muylaert et al., 2022; Simons et al., 2019; Slatculescu et al., 2020). In the present study, species distribution models improved our understanding of the potential for spatial overlap between invasive waterfowl and poultry in Arkansas. Regions with high levels of poultry production, most importantly northwest and central Arkansas, are also highly suitable for the invasive Egyptian Goose and Mute Swan, both of which host pathogens dangerous to poultry. Given the growing prevalence of poultry production globally, and especially throughout the southeastern United States, efforts to identify similar regions of overlap may be warranted as at least one of these invasive species, the Egyptian Goose, is growing in abundance in their invaded range.

## AUTHOR CONTRIBUTIONS

**Reilly T. Jackson:** Conceptualization (equal); data curation (lead); formal analysis (lead); investigation (equal); methodology (equal); writing – original draft (lead); writing – review and editing (equal). **Percival M. Marshall:** Conceptualization (equal); data curation (equal); investigation (equal); methodology (equal); writing – original draft (equal); writing – review and editing (equal). **Chris Burkhart:** Conceptualization (equal); data curation (equal); formal analysis (supporting); investigation (equal); methodology (equal); writing – original draft (supporting); writing – review and editing (equal). **Julia Schneck:** Conceptualization (supporting); data curation (supporting); writing – original draft (supporting); writing – review and editing (equal). **Grant Kelly:** Conceptualization (supporting); data curation (supporting); writing – original draft (supporting); writing – review and editing (equal). **Caleb P. Roberts:** Conceptualization (equal); formal analysis (supporting); funding acquisition (lead); investigation (supporting); methodology (supporting); project administration (equal); resources (lead); supervision (lead); writing – review and editing (equal).

## FUNDING INFORMATION

Funding was provided by the University of Arkansas Department of Biological Sciences and the Arkansas Game and Fish Commission through cooperative agreement 1434‐04HQRU1567.

## CONFLICT OF INTEREST STATEMENT

The authors have no conflicts of interest to declare.

## Supporting information


Data S1

## Data Availability

All data used in this study are publicly available via eBird (Mute Swan and Egyptian Goose presence data; https://science.ebird.org/en/use‐ebird‐data; eBird Basic Dataset, 2022), the Arkansas Highway and Transportation Department (chicken house data; https://gis.arkansas.gov/product/chicken‐house‐point/; AHTD, 2014), the WorldClim V2 Database (bioclimatic data; www.worldclim.org; Fick & Hijmans, 2017) and the 2019 Copernicus Global Landcover dataset (landcover data; https://developers.google.com/earth‐engine/datasets/catalog/COPERNICUS_Landcover_100m_Proba‐V‐C3_Global; Buchhorn et al., 2020).
