# Supplementary material for: Risk of invasive waterfowl interaction with poultry production: Understanding potential for avian pathogen transmission via species distribution models
Source: Ecol Evol. 2024 Jul 18;14(7):e11647. doi: 10.1002/ece3.11647 (PMC11257698; doi:10.1002/ece3.11647)
Supplement: Supplementary file 1 — Data S1 [file ECE3-14-e11647-s001.docx]

**SUPPLEMENTARY MATERIALS**

**Article Title**: Risk of invasive waterfowl interaction with poultry production: understanding potential for avian pathogen transmission

**Journal name**: *Ecology and Evolution*

**Authors**: Reilly T. Jackson^1*α^, Percival M. Marshall^1^, Chris Burkhart^1^, Julia Schneck^1^, Grant Kelly^1^, Caleb P. Roberts^2^

1. Department of Biological Sciences, University of Arkansas, Fayetteville, Arkansas, USA 72701
2. U.S. Geological Survey, Arkansas Fish and Wildlife Cooperative Research Unit, University of Arkansas, Fayetteville, Arkansas, USA 72701

*corresponding author: rtj006@uark.edu

^α^Current address: Wildlife Research Branch, Arizona Game and Fish Department, Phoenix, Arizona, USA 850865

Any use of trade, firm, or product names is for descriptive purposes only and does not imply endorsement by the U.S. Government.


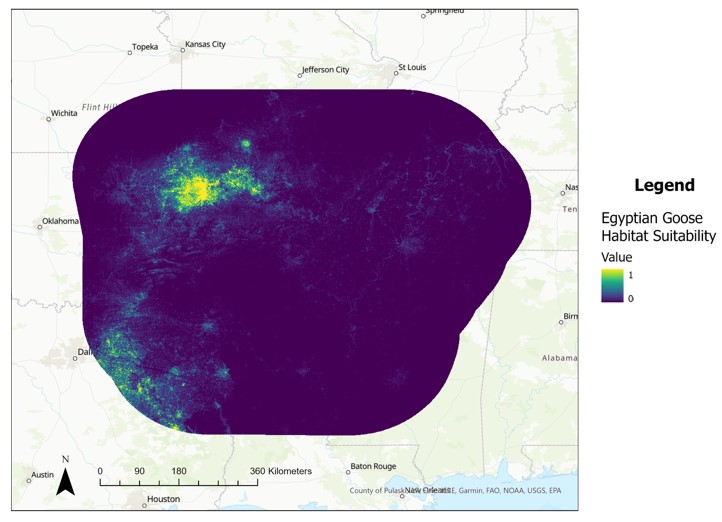


**Fig. S1** The full Maximum Entropy program (MaxEnt) output of the distribution of Egyptian Goose (*Alopochen aegyptiaca*) within 200 kilometers of the state of Arkansas’s border. Cell values are on a scale of 0-1, with 1 indicating 100% chance of presence and 0 indicating 0% chance of presence.
